# Supplementary material for: Development of a Dual-Readout Multicolor Immunoassay for the Rapid Analysis of Isocarbophos in Vegetable and Fruit Samples
Source: Foods. 2024 Dec 16;13(24):4057. doi: 10.3390/foods13244057 (PMC11675379; doi:10.3390/foods13244057)
Supplement: Supplementary file 1 [file foods-13-04057-s001.zip › foods-3363328-supplementary.pdf]

Development of a Dual-Readout Multicolor Immunoassay for the Rapid Analysis of Isocarbophos in Vegetable and Fruit Samples

Zijian Chen [2,3,4], Wei-Xuan Huang [5], Hongwu Wang [2,3,4], Meiling Zhang [2], Kai Chen [2] and Hao Deng [1,*]

[1] Key Laboratory of Tropical Fruit and Vegetable Cold-Chain of Hainan Province, Institute of Agro-Products of Processing and Design, Hainan Academy of Agricultural Sciences, Haikou 571100, China

[2] School of Food & Pharmaceutical Engineering, Zhaoqing University, Zhaoqing 526061, China; chenzijian@zqu.edu.cn (Z.C.); hwwang@zqu.edu.cn (H.W.); zml13430117492@163.com (M.Z.); blacoolker@163.com (K.C.)

[3] Laboratory of Quality & Safety Risk Assessment for Agro-Products (Zhaoqing), Ministry of Agriculture and Rural Affairs, Zhaoqing 526061, China

[4] Guangdong Engineering Technology Research Center of Food & Agricultural Product Safety Analysis and Testing, Zhaoqing 526061, China

[5] Department of Electrical Engineering, City University of Hong Kong, Hong Kong 999077, China; weixhuang6-c@my.cityu.edu.hk

***** Correspondence: denghao@hnaas.org.cn

Table S1 The gradient elution parameters of HPLC

Time (s)	Volume of phases A (%)	Volume of phases B (%)
0	97	3
1	97	3
1.5	85	15
2.5	50	50
18	30	70
23	2	98
27	2	98
27.1	97	3
30	97	3

Table S2 Comparison of immunoassay for isocarbophos analysis

Method	Mode	IC_{50}	Linear Range	LOD	Reference
ICA	Nake-eye analysis	ND[a]	ND	100 ng/mL	[1]
ICA	Nake-eye analysis	ND	ND	20 ng/mL	[2]
ELISA	Absorbance	58.85 ng/mL	ND	8.65 ng/mL	[3]
ELISA	Absorbance (two-step ELISA)	184.7 ng/mL	58.4-584.2 ng/mL	29.8 ng/mL	[4]
	Absorbance (one-step ELISA)	261.7 ng/mL	52.3-1309.6 ng/mL	20.4 ng/mL	
Immunosensor	Photothermal	ND	41.15-10000 ng/mL	2.62 pg/mL	[5]
	Ratiometric fluorescence	ND	0.24-7.81 ng/mL	0.21 pg/mL	
Multicolor immunoassay	Absorbance	11.5 ng/mL	3.9-62.5 ng/mL	4.6 ng/mL	This work
	RGB analysis	31.8 ng/mL	7.8-125 ng/mL	10.9 ng/mL	

[a]ND: No data

References

1. Wang, L.M.; Cai, J.; Wang, Y.L.; Fang, Q.K.; Wang, S.Y.; Cheng, Q.; Du, D.; Lin, Y.H.; Liu, F.Q. A bare-eye-based lateral flow immunoassay based on the use of gold nanoparticles for simultaneous detection of three pesticides. *Microchim. Acta* **2014**, *181*, 1565-1572.

2. Liu, B.Y.; Tang, Y.; Yang, Y.X.; Wu, Y.G. Design an aptamer-based sensitive lateral flow biosensor for rapid determination of isocarbophos pesticide in foods. *Food Control* **2021**, *129*, 108208.

3. Wang, C.M.; Li, X.B.; Liu, Y.H.; Guo, Y.R.; Xie, R.; Gui, W.J.; Zhu, G.N. Development of a Mab-Based Heterologous Immunoassay for the Broad-Selective Determination of Organophosphorus Pesticides. *J. Agr. Food Chem.* **2010**, *58*, 5658-5663.

4. Chen, Z.J.; Huang, Z.C.; Sun, Y.M.; Xu, Z.L.; Liu, J. The Most Active Oxidase‐Mimicking Mn_2O_3 Nanozyme for Biosensor Signal Generation. *Chemistry ‐ A European Journal* **2021**, *27*, 9597-9604.

5. Liu, J.; Deng, H.; Jia, B.Z.; Lin, Z.S.; Wang, Y.; Wang, H.; Xu, Z.L.; Luo, L. Ratiometric fluorescence and photothermal dual-mode immunosensor based on MnO2 nanosheets for the detection of isocarbophos. *Chem. Eng. J.* **2024**, *502*, 157951.
